# Polysaccharides Obtained from *Cordyceps militaris* Alleviate Hyperglycemia by Regulating Gut Microbiota in Mice Fed a High-Fat/Sucrose Diet

**DOI:** 10.3390/foods10081870

**Published:** 2021-08-12

**Authors:** Bao-Hong Lee, Chia-Hsiu Chen, Yi-Yun Hsu, Pei-Ting Chuang, Ming-Kuei Shih, Wei-Hsuan Hsu

**Affiliations:** 1 Department of Horticulture, National Chiayi University, Chiayi 600355, Taiwan; bhlee@mail.ncyu.edu.tw; 2 Department of Food Safety/Hygiene and Risk Management, College of Medicine, National Cheng Kung University, Tainan 701401, Taiwan; hebe12311@gmail.com (C.-H.C.); llily333555tw@gmail.com (Y.-Y.H.); 3 Institute of Food Safety and Risk Management, National Taiwan Ocean University, Keelung 202301, Taiwan; ptchuang@mail.ntou.edu.tw; 4 Graduate Institute of Food Culture and Innovation, National Kaohsiung University of Hospitality and Tourism, Kaohsiung 812301, Taiwan; mkshih@mail.nkuht.edu.tw; 5 Center of Allergy and Mucosal Immunity Advancement, National Cheng Kung University, Tainan 701401, Taiwan

**Keywords:** *Akkermansia*, *Cordyceps militaris*, metabolic syndrome, gut microbiota, polysaccharides

## Abstract

Polysaccharides isolated from fungus *Cordyceps militaris* display multi-biofunctions, such as immunostimulation, down-regulation of hyperlipidemia, and anti-cancer function. The occurrence of obesity and metabolic syndrome is related to the imbalance of gut microbiota. In this study, the effects of *C. militaris* and its fractions on modifying metabolic syndrome in mice were evaluated. Mice were fed a high-fat/high-sucrose diet (HFSD) for 14 weeks to induce body weight increase and hyperlipidemia symptoms in mice, and then the mice were simultaneously given a HFSD and *C. militaris* samples for a further 8 weeks. The results indicated that the fruit body, polysaccharides, and cordycepin obtained from *C. militaris* had different efficacies on regulating metabolic syndrome and gut microbiota in HFSD-treated mice. Polysaccharides derived from *C. militaris* decreased the levels of blood sugar and serum lipids in mice fed HFSD. In addition, *C. militaris*-polysaccharide treatment obviously improved intestinal dysbiosis through promoting the population of next generation probiotic *Akkermansia muciniphila* in the gut of mice fed HFSD. In conclusion, polysaccharides derived from *C. militaris* have the potential to act as dietary supplements and health food products for modifying the gut microbiota to improve the metabolic syndrome.

## 1. Introduction

Mushroom *Cordyceps militaris* is a widely used fungus for medicinal purposes. It has been found to inhibit tumor growth [1], leukemia [2], liver cancer [3], kidney cancer [4], and oral cancer [5]. Cordycepin, also called 3′-deoxyadenosine, is a purine nucleoside isolated from *C. militaris*, which has physiological activities and is considered to be the main anti-cancer substance in *C. militaris*. Our past research has found that in the process of liquid culture of *C. militaris*, different fermentation substrates not only affect the yields of biomass production (including extracellular polysaccharides, cordycepin, and mycelium production), but also affect the activity of suppressing oral cancer [6]. In addition to cancer suppression, *C. militaris* also has the potential to improve metabolic syndrome, including diabetes [7], hypertension blood pressure [8], and hyperlipidemia [9]. There are more than 1000 different microbial species (species) in the human gastrointestinal (GI) tract; these bacterial phases constitute a specific microenvironment in the gut. A study highlighted that different microbial ecology will affect the body’s biochemical function, heredity, and disease occurrence [10]. Gut microbiota have genes that encode over 4,000,000 proteins and cause biological responses to the human body [11]. In the human GI tract, the small intestine is considered to contain the largest number of microorganisms (mostly anaerobic or facultative anaerobes), the most complex types of bacteria, and it is susceptible to changes in foreign nutrients or dietary conditions, while the ileum and large intestine (colon) are distributed with a large number of anaerobic microorganisms [12]. High-protein or high-fat diets will promote the ratio of the phylum of *Bacteroides* in the intestine, whereas the ratio of phylum *Prevotella* is greatly increased due to the large amount of carbohydrates, plant polysaccharides, or glycoproteins in the diet. Moreover, the microbes of the *Ruminococcus* phylum use mucins as a nutrient source and help intestinal cells absorb glucose, showing that gut microbiota can affect the activity and physiological functions of intestinal cells such as enterocytes [13,14]. Since metabolic diseases are related to gut microbiota dysbiosis, the study found that mice with high hyperlipidemia were accompanied by a large amount of genus *Desulfovibrio* in the intestines [15], and another study has found that high-fat diet leads to the increase of pathogenic *Alistipes* and *Anaerotruncus*, as well as induces decreases in *Lactobacillus* and *Alloprevotella* in mice [16].

Recently, the cordycepin and *Cordyceps*-derived polysaccharides have been found to attenuate microbiota dysfunction and improve obesity in rat and mice [17,18]. Many experiments have found that *Cordyceps*-derived polysaccharides have many physiological regulation potentials, including preventing damage of intestinal cells [19] and promoting immunostimulatory activity [20]. In addition, the monosaccharide composition and structure of *Cordyceps*-derived polysaccharides have also been studied [21], and their adhesive properties have been explored to be used in the maintenance of starch stability [22]. The purpose of this study is to investigate the regulatory effects of *C. militaris* fruit body, *C. militaris*-derived polysaccharides, and cordycepin-rich solution on gut microbiota in mice treated with a high-fat diet, and to evaluate the potentials of *C. militaris*-derived polysaccharides as a healthy food for improving metabolic syndrome.

## 2. Materials and Methods

### 2.1. Chemicals

Adenosine and cordycepin were purchased from Sigma Chemical Co. (St Louis, MO, USA). The hematoxylin and eosin (HE) stain kit (CATA: ab245880) and periodic acid Schiff (PAS) stain kit (CATA: ab150680) were purchased from Abcam (Cambridge, MA, USA).

### 2.2. Sample Preparation

After crushing the fruit body of *C. militaris*, it was extracted with hot water (1:10/*w*:*w*) for 1 h, and then filtered with filter paper. After cooling the supernatant, anhydrous alcohol was added to precipitate the polysaccharide (1:10/*v*:*v*) and the mixture was centrifuged at 5000 rpm (4 °C). The precipitate and supernatant were divided into the crude polysaccharide and cordycepin-rich solution, respectively. These materials were treated with vacuum concentration and freeze-dried, hence stored at −80 °C. *Bacillus coagulans* IF01 strain was cultured with nutrient broth and grown under aerobic conditions at 37 °C for 24 h. The number of bacteria were counted by aerobic cultivation on nutrient agar plates. *B. coagulans* IF01 was obtained in our previous work, which was isolated from infant feces and has the properties of lactic acid production. Although *B. coagulans* species is Generally Recognized as Safe (GRAS) for its intended use, this is the first study to demonstrate the beneficial effect of the *B. coagulans* IF01 strain on improving the metabolic syndrome. 

### 2.3. High Performance Liquid Chromatography (HPLC) Assay for Cordycepin Level

The cordycepin-rich supernatant was filtered by 0.45-μm membrane, and the cordycepin and adenosine levels were measured by HPLC analysis with RP-18 column modifying the method of Chang, Lue, and Pan (2005) [23]: the mobile phase was a mixture of methanol and deionized water, the column temperature was 40 °C, the flow rate was 1.0 mL/min, and the detector was set at 260 nm. 

### 2.4. Western Blotting

Ice-cold buffer containing 1% of Triton X-100, 0.1% of SDS, 500 mM of sodium vanadate, 20 mM of Tris-HCl (pH 7.4), 10 mM of NaF, 2 mM of EDTA, 1 mM of phenyl-methanesulfonyl fluoride, and 10 mg/mL of aprotinin was used to lyse the cells. Supernatant of cells was obtained from the centrifuged (12,000× *g*, 10 min) cell lysate. SDS-PAGE (10%) was used to resolve the proteins and transfer them to a polyvinylidene fluoride membrane. Non-fat milk (5%) was used to block membranes for 1 h and then primary antibodies were added to membranes for 2–4 h. Subsequently, the membrane was washed with phosphate-buffered saline with Tween-20 (PBST) for 5 min for three times and incubated with horseradish peroxidase (HRP)-linked secondary antibody for 1 h. After washing three times with PBST, the enhanced chemiluminescent reagent (Millipore, Billerica, MA, USA) was used to determine the protein concentration. 

### 2.5. Animal Experiment

Experimental animals used in this study had been reviewed and approved by the Institutional Animal Care and Use Committee (IACUC) in National Cheng Kung University in Taiwan with IACUC approval No. 108317. The 6-week-old C57BL/6 male mice were subjected to a high-fat/high-sucrose diet (HFSD) to induce body weight increased and hyperlipidemia symptoms in mice. The animals were treated with a diet containing 40% fat and 45% sucrose for 14 weeks, and then the mice were simultaneously given a HFSD and *C. militaris* or its different fraction; each sample was dissolved in 0.2 mL of sterile water and given by oral gavage with a 22 G needle once a day for another 8 weeks. Mice in the control group were fed a normal diet and gavaged with 0.2 mL of sterile water once a day. Blood and stool of mice were collected at the end of the 14th week. All animals were weighed and their data recorded every 2 weeks during the experiment. At the end of the experiment, the mice were deprived of food for 12 h and were euthanized with carbon dioxide. Feces, blood, liver tissue, and intestinal tissue were sampled. Blood samples were collected from the posterior vena cava and centrifuged at 700× *g* for 10 min, and serum was stored at −20 °C until analyzed.

### 2.6. Assays for Serum Triglyceride and Total Cholesterol

Serum total cholesterol (TC) and triglyceride (TG) commercial kits (CH 200 for TC, TR-210 for TG) were obtained from Randox Laboratories Ltd., (Antrim, UK), and the measurement for serum TC and TG were carried out according to the manual.

### 2.7. The Hematoxylin and Eosin (H&E) Stain

Mice were sacrificed and the colons were trimmed into 2-mm thickness and fixed in 10% neutral buffered formalin. After fixation, the tissue was embedded in paraffin (5-μm) and H&E stain was carried out according to the manual.

### 2.8. Periodic Acid Schiff (PAS) Stain

The PAS stain kit is determined for the histological identification of lymphocytes and mucopolysaccharides. Colon sections of mice were hydrated in distilled water and the slides were immersed in Periodic Acid Solution for 5–10 min. Then, the slides were rinsed in 4 changes of dH_2_O and then immersed in Schiff’s Solution for 15–30 min. The slides were further rinsed in hot running tap water and subsequently rinsed in dH_2_O. Hematoxylin (Modified Mayer’s) was used to stain the slides for 2–3 min. After rinsing in running tap water for 2–3 min, the slides were applied to Bluing reagent for 30 s and then rinsed in dH_2_O. The slides were further dehydrated by graded ethanol and cleared, then mounted in synthetic resin.

### 2.9. Assay for Gut Microbiota

Feces in the colon of each mouse were collected and immediately soaked in liquid nitrogen and stored at −80 °C for subsequent use. Total genomic DNA from samples was extracted using QIAamp PowerFecal DNA Kit (Qiagen) for full-length 16S rDNA amplicon sequencing by using LoopSeq™ 16S Microbiome SSC kit and Illumina Sequencing-by-Synthesis (SBS) technology. High quality consensus contigs were then used for species-level classification using Loop Genomics cloud platform. Contigs were further filtered by percentage of coverage to reference sequence and further filtered by alignment identity to ensure the accuracy that the final contigs had both high coverage and high identity to reference sequences.

### 2.10. Statistical Analysis

Experiments were performed in five repeats and the results are expressed as the mean ± standard error of mean. The results were examined by using one-way analysis of variance and Duncan’s multiple range tests, and the significance of differences between sample means was calculated. A *p* value ≤ 0.05 was considered significant. For β diversity of microbiota analysis, Pairwise ANOSIM (Analysis of similarities) with 999 permutations were conducted and evaluated using Principal coordinate analyses (PCoA) based on different distance matrices, where *p* values were reported after Benjamini–Hochberg multiple testing correction (*q* value).

## 3. Results and Discussion

### 3.1. Analyzing the Levels of Cordycepin in C. militaris

**Figure 1:**
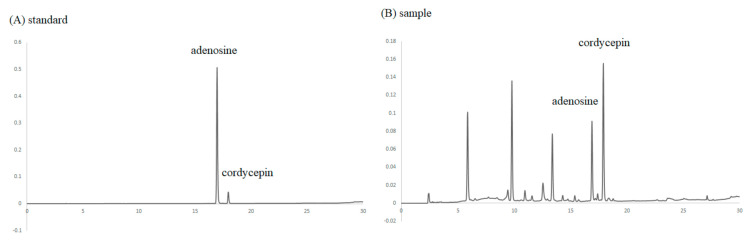
HPLC analysis of adenosine and cordycepin in the cordycepin-rich supernatant. Chromatogram of (**A**) standard and (**B**) cordycepin-rich supernatant.

Both cordycepin and adenosine are components of *C. militaris*. Adenosine is an endogenous purine nucleoside and is the precursor of cordycepin, which acts as a cofactor during cordycepin bioconversion. After re-dissolving the cordycepin-rich supernatant with deionized water (1:10/*w*:*w*), the contents of adenosine and cordycepin were analyzed by HPLC. The profile is shown in Figure 1, and the results show that it contains 105 μg/g of adenosine and 93.7 μg/g of cordycepin. Other peaks in the spectrum indicated that the layer of cordycepin-rich supernatant contained certain components, and it remains for these to be explored in the future.

### 3.2. The Effects of C. militaris on Lowering Blood Glucose, TG, and TC Levels in High Fat/Sucrose Diet (HFSD)-Induced Mice

**Figure 2:**
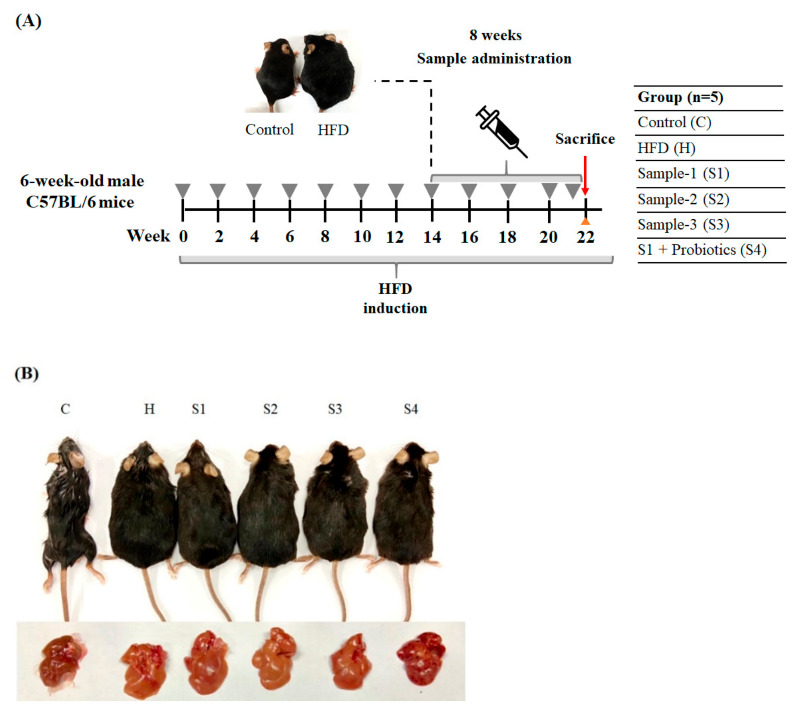
The effects of *C. militaris* on hyperglycemia in mice fed a high-fat/sucrose diet. (**A**) Schematic diagram of research design. (**B**) The appearance of body shape and liver in mice.

The period of HFSD inducing in mice and feeding samples is shown in Figure 2A. After 14 weeks of HFSD induction, samples were orally given to mice for 8 weeks. The sample feeding groups included feeding the fruit body of *C. militaris* (S1), *C. militaris*-derived polysaccharides (S2), cordycepin-rich supernatant (S3), and *C. militaris* fruit body combined with probiotic (*B. coagulans* IF01) (S4). After the dosage calculating conversion, approximately 40 μg/kg bw per day of cordycepin was intake in HFSD-induced mice administered with the cordycepin-rich supernatant. In addition, *B. coagulans* has been proven to improve the intestinal tract, reduce body fat production, and regulate blood glucose [24]. Since *B. coagulans* has been found to show the potential for hydrolyzing polysaccharides [25], this ability may allow this probiotic using *C. militaris*-derived polysaccharides as a nutrient source. This study intended to evaluate *C. militaris* fruit body synergistically with *B. coagulans* to improve blood glucose and gut microbiota in mice induced by HFSD. In the feeding group of *C. militaris* fruit body with probiotic, the feeding concentrations of *B. coagulans* was 10^10^ CFU/mouse in this study. The appearance of the body shape and liver in mice after being sacrificed is shown in Figure 2B. It suggests that HFSD induction markedly resulted in obesity and fat accumulation in the liver.

**Figure 3:**
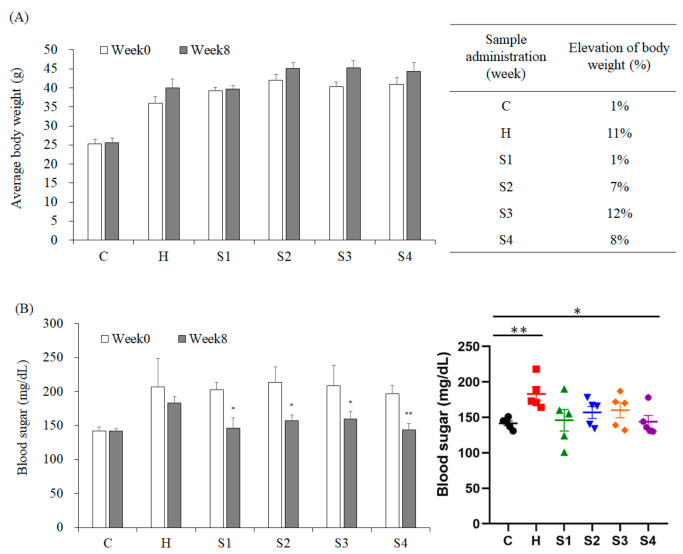
The effects of *C. militaris* on body weight and blood sugar in mice fed a high-fat/sucrose diet. (**A**) Total body weight and body weight gain of mice after treating with samples for 8 weeks. (**B**) The levels of blood sugar in the serum of high-fat/sucrose diet-fed mice after 8-week treatment. Data are shown as means ± SEM (*n* = 5). * *p* < 0.05, ** *p* < 0.01 compared to the control group.

**Figure 4:**
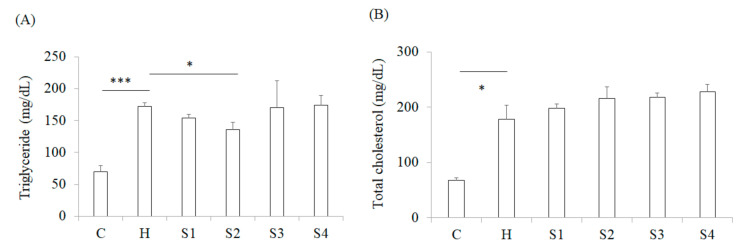
The effects of *C. militaris* on serum lipids of high-fat/sucrose diet-fed mice. Serum triglyceride (**A**) and total cholesterol (**B**) levels in mice after treating with samples for 8 weeks. Data are shown as means ± SEM (*n* = 5). * *p* < 0.05, *** *p* < 0.001 compared to the H group.

The results (from week-0 feeding with samples to week-8) revealed that HFSD induction significantly increased the body weight of mice by 11%, and feeding with *C. militaris* fruit body effectively inhibited the weight gain of mice (S1 group), whereas this effect was not observed in group S4. This situation implied that the polysaccharides of *C. militaris* fruit body could have been decomposed by *B. coagulans*, and lead to the disappearance of suppressing the body weight gain of the mice. Moreover, comparing with the S1 group, it was found that *C. militaris*-derived polysaccharide (S2 group) also had the ability to inhibit weight gain, but this activity was not found in mice fed cordycepin-rich supernatant (S3 group) (Figure 3A). The in vivo results (from week-0 feeding with samples to week-8) revealed that HFSD induction significantly increased blood glucose level in mice; however, the administration of samples could significantly reduce blood glucose (Figure 3B). In addition, only *C. militaris*-polysaccharides feeding could significantly reduce the TG concentration in the serum of HFSD-induced mice, while the cordycepin-rich supernatant, *C. militaris* fruit body, with or without probiotics, could not reduce the TG concentration (Figure 4A). Serum TC content is also an evaluation indicator for metabolic syndrome. It was found that HFSD induction significantly increased the serum TC level in mice, but no serum TC concentration of mouse was decreased due to the sample administration (Figure 4B). These results indicate that the ability of *C. militaris* polysaccharide for the regulation of blood glucose was better than its ability for the regulation of hyperlipidemia. Many plant- and fungal-polysaccharides have been found to prevent oxidative stress and promote insulin sensitivity, thereby improving the symptoms of hyperglycemia in diabetic animals, including *Ganoderma lucidum* [26], *Solanum lycocarpum* fruit [27], *Dioscorea batatas* [28], and *Lycium barbarum* [29].

### 3.3. The Protection of C. militaris on Intestinal Function in HFSD-Induced Mice

**Figure 5:**
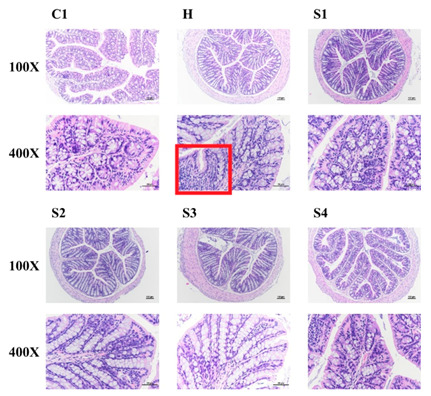
The effects of *C. militaris* on the colon of mice fed a high-fat/sucrose diet. Representative Hematoxylin and eosin (H&E) stained colon sections are shown.

**Figure 6:**
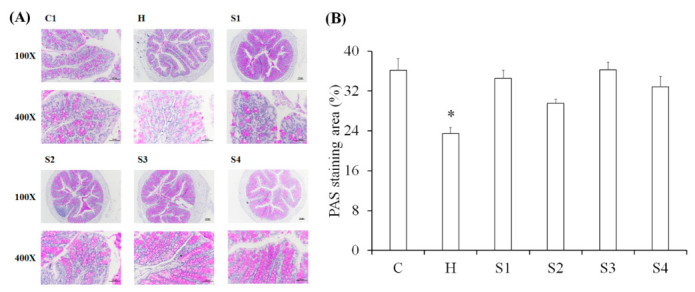
Periodic acid-Schiff staining (PAS) staining in the colon of mice. (**A**) Representative PAS stained sections. (**B**) Quantification of PAS staining. Data are shown as means ± SEM (*n* = 5). * *p* < 0.05 compared to the control group.

After staining of HE sections, it was found that HFSD induction increased the infiltration of immune cells in the intestine. However, this intestinal inflammation caused by HFSD induction was not found in other sample feeding groups (Figure 5). The PAS could stain for intestinal mucus and the results are shown in Figure 6. Results indicated that HFSD induction markedly reduced intestinal mucus level in mice, but *C. militaris* fruit body, *C. militaris*-polysaccharide, and cordycepin-rich supernatant could improve intestinal mucus production in HFSD-induced mice. However, the situation of feeding *C. militaris* fruit body to increase the production of intestinal mucus could not be found in the *C. militaris* fruit body with probiotic-treated group (S4). It is speculated that *B. coagulans* may degrade the polysaccharides in the fruit bodies of *C. militaris* or decompose intestinal mucus. When pathogens invade the host, the mucosal surface in the gastrointestinal tract can block pathogens entering into the blood from the intestine. Therefore, these mucosal surfaces contain a lot of mucus which is a physical flow barrier. In addition, the intestinal epithelial cells in the tract secrete many defense compounds into the mucosa, thereby increasing the ability to inhibit pathogenic bacteria [30]. Studies have shown that mucin production is increased when intestinal cells are exposed to microbial metabolites. For example, the virulence factors produced by *Helicobacter pylori* and *Campylobacter jejuni* have been shown to attach to intestinal epidermal cells. These virulence factors also regulate mucin production while pathogens infect the host [31]. In addition, some short-chain fatty acids up-regulated the ability of intestinal cells for mucin production, such as butyrate which can increase the expression of mucin mRNA [32]. Some microorganisms (*Ruminococcus* and *Dorea*) within the family *Lachnospiraceae* are able to use intestinal mucin as a nutrient source for bacterial growth [33].

A large number of microorganisms that degrade mucus are distributed in the lumen of the large intestine, including *Akkermansia muciniphila* [34], *Bacteroides thetaiotaomicron* [35], *Bifidobacterium bifidium* [36], *Bacteroides fragilis* [37], and *Ruminococcous gnavus* [38]. These bacteria will degrade mucus O-glycans and produce monosaccharides [39], allowing other mucus decomposing bacteria to further utilize these mucus residues for growth. This group of microorganisms that decompose mucus residues are mainly *Lachnospiraceae* [30], *Clostridium* cluster XIV [40], *Enterbacteriaceae* [41], and *Clostridium difficile* [42]. The intestinal microenvironment will change after mucus degradation; subsequently, *Lactobacillus* [43] and *Bacteroides* [44] can adapt to this microenvironment and begin to proliferate. Moreover, microorganisms in the *Prevotella* phylum will increase greatly due to the large amount of carbohydrates, plant-based polysaccharides, or glycoproteins in the diet; microorganisms in the *Ruminococcus* phylum will use mucins as a source of nutrients and help the glucose uptake of intestinal cells [13,14]. Our research results have revealed that the production of intestinal mucus is inhibited by HFSD induction, but *C. militaris*-polysaccharides can improve the production of mucus, indicating that *C. militaris*-polysaccharides should be able to regulate the intestinal microbiota. This study will next explore the regulatory effect of *C. militaris*-polysaccharides on intestinal microbes, as well as the relationship between these microbes and HFSD-induced metabolic disorders.

### 3.4. The Regulation of C. militaris on Gut Microbiota in HFSD-Induced Mice

**Figure 7:**
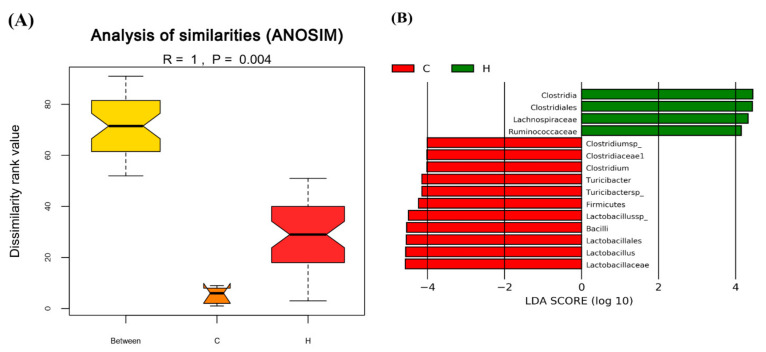
Statistical analysis and biomarkers of gut microbiota in control and high-fat/sucrose diet-fed mice after 14 weeks of induction. (**A**) Analysis of similarities (ANOSIM) method was used to determine the significance of differences in the species composition and community results of the grouped samples. Results based on Bray–Curtis dissimilarities (*p* = 0.004). (**B**) Linear discriminant analysis Effect Size (LEfSe) was used to estimate the magnitude of the impact of each species abundance on the difference effect, and find the communities or species that have significant differences in the sample division. The Linear discriminant analysis (LDA) distribution histogram shows the species as biomarkers whose LDA Score is greater than the threshold setting value (default: 4) with statistical differences between groups. The length of the histogram represents the impact of different species. Results based on taxonomy data.

**Figure 8:**
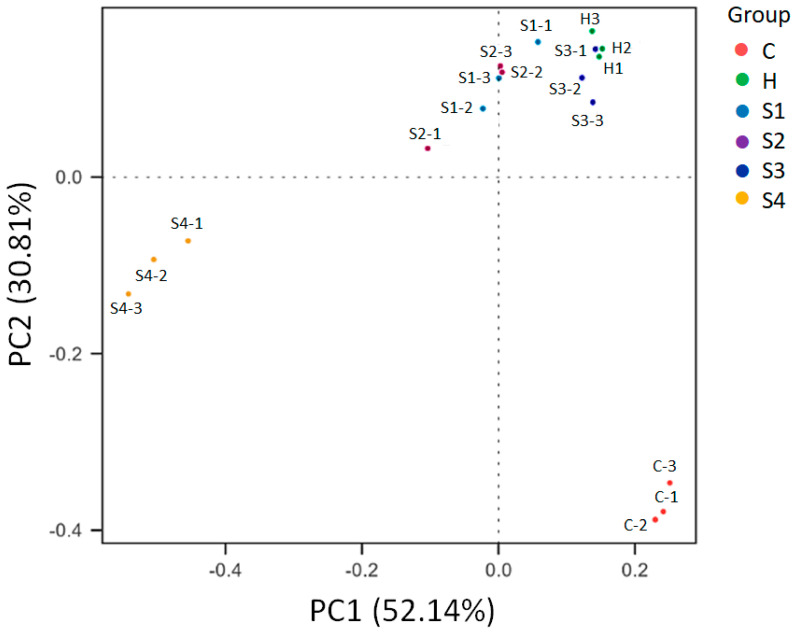
The effects of *C. militaris* on gut micriobiota distribution in mice fed a high-fat/sucrose diet. Principal Coordinates Analysis (PCoA) was used to determine beta diversity of gut microbiota. Samples with more similar compositions cluster closer together.

Before the administration of samples to the mice, they were given HFSD for 14 weeks. After 14 weeks of HFSD induction, the feces of mice in the control group (group C) and HFSD treatment group (group H) were collected and the composition of gut microbiota was analyzed. The data in Figure 7 show the statistical analysis and biomarkers of gut microbiota between the control group (group C) and HFSD treatment group (group H). Non-parametric statistical method analysis of similarities (ANOSIM) was used to determine the significance of differences in the species composition and community results of the grouped samples to understand whether the difference between groups was significantly greater than the difference within the group, so as to judge whether the grouping was meaningful [45]. The results were based on Bray–Curtis dissimilarities and there was a significant difference between the two groups (*p* = 0.004) (Figure 7A), indicating that gut microbiota composition was markedly changed in mice after HFSD induction. We also used the statistical Linear discriminant analysis Effect Size (LEfSe) method to determine the significance of differences in the species composition and community results of the grouped samples. LEfSe uses the non-parametric factorial Kruskal–Wallis (KW) sum-rank test to clarify species with significant differences in abundance, and uses linear regression discriminant analysis (LDA) to estimate the impact of each species abundance on the difference effect to find out communities or species affected by significant differences in sample division [46]. The results in Figure 7B show the LDA distribution histogram, the species as biomarkers whose LDA score is greater than the threshold setting value (default: 4) with statistical differences between groups. After HFSD induction, the levels of the genus *Lactobacillus* were obviously dropped when compared to the control group. The data of Beta diversity analysis of gut microbiota using the Principal Coordinates Analysis (PCoA) is shown in Figure 8. The PCoA is a dimension reduction technique used to express multivariate data in fewer dimensions. The abscissa indicates the first principal component, the ordinate indicates the second principal component, and the percentage indicates the contribution rate of the principal component to the sample difference. Each plot in Figure 8 indicates one sample, and the same grouped samples are represented by the same color. Samples with more similar compositions cluster closer together. The data showed that the composition of gut microbiota between the control and HFSD induction groups had a greater degree of dispersion, and the group administered the samples were also far from control or HFSD, especially the S4 group, which had the greatest degree of dispersion from the control and HFSD induction groups. The possibility that the probiotic has a potential to affect the composition of gut microbiota in mice exists. 

**Figure 9:**
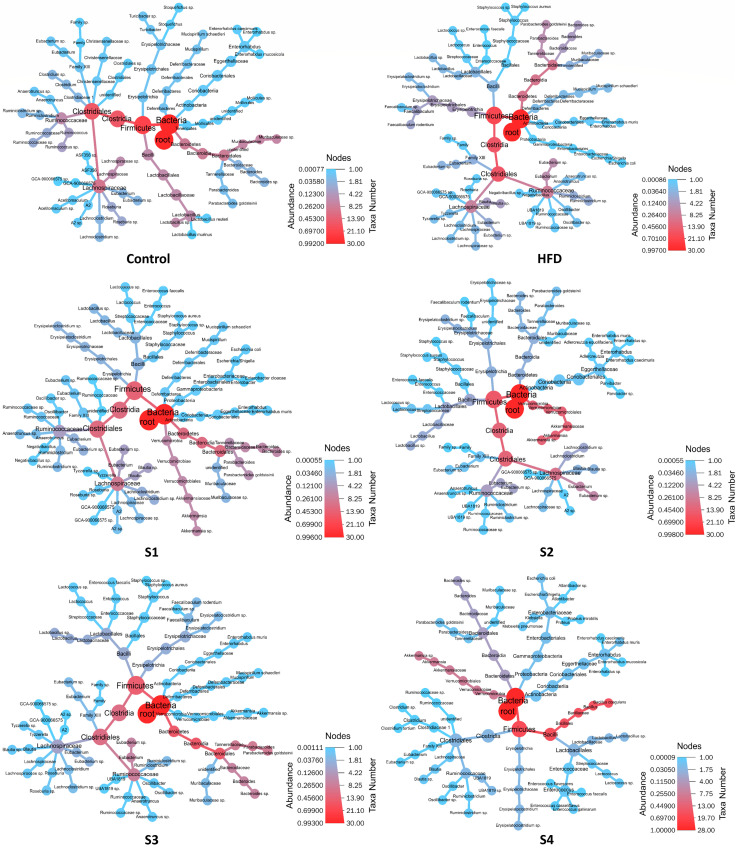
The effects of *C. militaris* on gut micriobiota composition in mice fed a high-fat/sucrose diet by Heat Tree plot. Differential taxonomic tree displaying the changes in taxa abundance between groups. Top 30 species for each group are shown.

After tested samples were given to mice for 8 weeks, feces of mice in each group were collected and gut microbiota analyzed by LoopSeq™ 16S sequencing. The top 30 species for each group are shown in Figure 9 by the Heat Tree plot. Differential taxonomic tree displays the changes in taxa abundance between groups. The circular heat tree represents the sequence abundance of different classes. From the center to the periphery of the heat tree, the displayed hierarchy is from Kingdom to Species. Sequence abundance is marked by node size, branch thickness, and color; the color of the species with higher abundance is closer to red. Figure 9 shows the relatively dominant species in each group.

**Figure 10:**
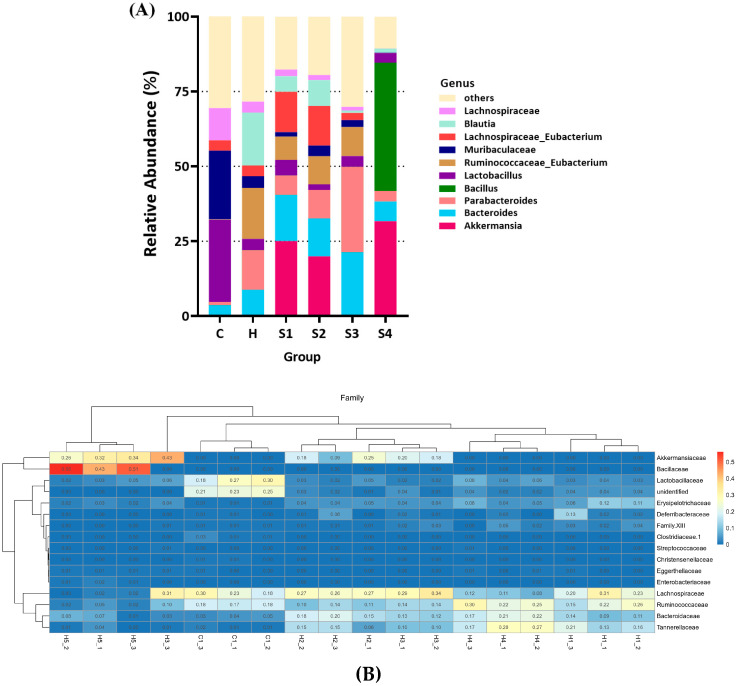
Taxa analysis of gut microbiota composition in mice. (**A**) Relative abundance bar chart. Top 10 classification for genus level for each group are shown. Taxa not in the top 10 are grouped together in the ‘others’ category. (**B**) Hierarchical clustering heatmap with dendrograms. Interactive hierarchical clustering heatmap represents relative abundance of taxa on a color gradient. The top 40 most abundant family taxa are shown.

In taxa analysis of gut microbiota composition, the data in Figure 10A show the relative abundance bar chart and top 10 classification for genus level for each group. Taxa not in the top 10 are grouped together in the “others” category. Probiotic treatment may have the potential to change the composition of gut microbiota in mice. The data showed that the main influence of *B. coagulans* treatment (group S4) was markedly increased the ratio of genus *Bacillus* in gut microbiota of mice, while other genus composition was similar to the group *C. militaris* fruit body (S1) and *C. militaris* polysaccharides (S2).

Figure 10B displays hierarchical clustering heatmap with dendrograms. The interactive hierarchical clustering heatmap represents the relative abundance of taxa on a color gradient. The top 40 most abundant family taxa are shown. The results showed that the genus *Blautia*, *Ruminococcaceae_Eubacterium*, *Parabacteroides*, and *Bacteroides* were obviously increased, but *Lactobacillus*, *Muribaculaceae*, and *Lachnospiraceae* were markedly reduced in the HFSD induction group when comparing with the control group. Although the limitation of the study is the lack of a control group with probiotic alone, interestingly, the cordycepin-rich supernatant clearly expanded the increases in elevations of *Bacteroides* and *Parabacteroides* caused by HFSD induction, showing that the cordycepin-rich supernatant (S3) cannot show the regulating effect of restoring the gut microbiota. On the other hand, *C. militaris* fruit body (S1), *C. militaris* polysaccharides (S2), and *C. militaris* fruit body with probiotics (S4) treatment effectively increased the populations of *Akkermansia* and *Lachnospiraceae_Eubacterium*, as well as decreasing *Bacteroides*, *Parabacteroides*, and *Blautia*, even through oral administration of *B. coagulans* would significantly increase the proportion of the *Bacillus* population in the S4 group. *A. muciniphila* is the only member of the genus *Akkermansia* spp. [47], which is a mucin degrading bacterium and is associated with beneficial health effects, such as reducing intestinal inflammation and improving gut barrier function [48]. Evidence also suggests that *Akkermansia* prefers to ingest carbohydrates (mainly polysaccharides) as its nutritional source [34]. *A. muciniphila* has the ability to improve diabetes, reducing body fat production, and suppressing weight gain; therefore, increasing *A. muciniphila* in the intestines through diet is believed to have the ability to improve metabolic syndrome [49,50]. As shown in Figure 10A, the relative abundances of *Akkermansia* spp. in group *C. militaris* fruit body (S1), *C. militaris* polysaccharides (S2), and *C. militaris* fruit body with probiotics (S4) were 17.2%, 26.9%, and 30.4%, respectively, whereas there were under 0.1% relative abundances of *Akkermansia* spp. in the control group (group C), HFSD treatment group (group H), and cordycepin-rich supernatant (S3). Analysis of these results indicated that *C. militaris* and its polysaccharides have the ability to improve intestinal dysbiosis and promote the growth of gut *Akkermansia* spp., which may include the next generation probiotic *Akkermansia muciniphila*. 

Lactic acid bacteria are also considered in regards to the fact that the *Lactobacillus* metabolites could relieve hyperglycemia in obese rat [51]. Microorganisms of the *Lachnospiraceae* family can be found in the intestines of mammals. All the bacteria in the *Lachnospiraceae* family are anaerobic bacteria, which are characterized by the production of a large amount of butyrate, which is considered to be a short-chain fatty acid that can inhibit opportunistic bacteria growth and prevent colorectal cancer [52]. Under *Lachnospiraceae* family, *Blautia* and *Roseburia* are representative microorganisms that control intestinal inflammation, atherosclerosis, and immune system activation [53]. In particular, *Blautia* has been found in many clinical studies to be effective in diseases such as colorectal cancer and liver cirrhosis through its anti-inflammatory effects [54,55]. Genus *Dorea* also plays an anti-inflammatory role in patients with non-alcoholic fatty liver [56]. It has also been found to contain a large number of microorganisms of the *Lachnospiraceae* family, especially *Blautia* and *Lachnospiracease incertaesedis* [57]. The abundance of *Lachnospiraceae* is positively correlated with glucose/lipid metabolism, hence this microorganism is considered to be an indicator of metabolic disorders [52]. The role of inflammation and pro-inflammation in *Lachnospiraceae* should be confirmed in the future. These studies show that the significance of *C. militaris* fruit body and *C. militaris*-polysaccharides in regulating *Lachnospiraceae* remains to be explored, but it is confirmed for *A. muciniphila*.

## 4. Conclusions

In this study, the effects of *C. militaris* on regulating metabolic syndrome in mice were evaluated. The results showed that the fruit body of *C. militaris*, the polysaccharides isolated from *C. militaris*, and cordycepin-rich solution obtained from *C. militaris* had different efficacies on regulating hyperglycemia and gut microbiota in HFSD-induced mice. Polysaccharides derived from *C. militaris* treatment improved intestinal dysbiosis through promoting the population of gut next generation probiotic *A. muciniphila* in mice fed HFSD. According to the above, *C. militaris*-derived polysaccharides have the potential to act as a dietary supplement and health food for improving the metabolic syndrome via intestinal regulation.

## References

[1] Yoshikaw N., Nakamura K., Yamaguchi Y., Kagota S., Shinozuka K., Kunitomo M. (2004). Antitumour activity of cordycepin in mice. Clin. Exp. Pharmacol. Physiol..

[2] Wehbe-Janek H., Shi Q., Kearney C.M. (2007). Cordycepin/hydroxyurea synergy allows low dosage efficacy of cordycepin in MOLT-4 leukemia cells. Anticancer Res..

[3] Chen Y., Chen Y.C., Lin Y.T., Huang S.H., Wang S.M. (2010). Cordycepin induces apoptosis of CGTH W-2 thyroid carcinoma cells through the calcium-calpain-caspase 7-PARP pathway. J. Agric. Food Chem..

[4] Xu H.L., Zhang L.J., Shi H., Zhu X., He X. (2014). Effects of cordycepin on Hep G2 and EA.hy926 cells: Potential antiproliferative, antimetastatic and anti-angiogenic effects on hepatocellular carcinoma. Oncol. Lett..

[5] Yamamoto K., Shichiri H., Uda A., Yamashita K., Nishioka T., Kume M., Makimoto H., NNakagawa T., Hirano T., Hirai M. (2015). Apoptotic effects of the extracts of *Cordyceps militaris* via Erk phosphorylation in a renal cell carcinoma cells line. Phytother. Res..

[6] Lin L.T., Lai Y.J., Wu S.C., Hsu W.H., Tai C.J. (2018). Optimal conditions for cordycepin production in surface liquid-cultured *Cordyceps militaris* treated with porcine liver extracts for suppression of oral cancer. J. Food Drug Anal..

[7] Sun H., Yu X., Li T., Zhu Z. (2021). Structure and hypoglycemic activity of a novel exopolysaccharide of *Cordyceps militaris*. Int. J. Biol. Macromol..

[8] Takakura K., Ito S., Sonoda J., Tabata K., Shiozaki M., Nagai K., Shibata M., Koike M., Uchiyama Y., Gotow T. (2017). *Cordyceps militaris* improves the survival of Dahl salt-sensitive hypertensive rats possibly via influences of mitochondrial and autophagy functions. Heliyon.

[9] Lee M.R., Kim J.E., Choi J.Y., Park J.J., Kim H.R., Song B.R., Choi Y.W., Kim K.M., Song H., Hwang D.Y. (2019). Anti-obesity effect in high-fat-diet-induced obese C57NL/6 mice: Study of a novel extract from mulberry (*Morus alba*) leaves fermented with *Cordyceps militaris*. Exp. Ther. Med..

[10] Aziz Q., Dore J., Emmanuel A., Guarner F., Quigley E.M.M. (2013). Gut microbiota and gastrointestinal health: Current concepts and future directions. Neurogastroenterol. Motil..

[11] Venter J.C., Adams M.D., Myers E.W., Li P.W., Mural R.J., Sutton G.G., Smith H.O., Yandell M., Evans C.A., Holt R.A. (2001). The sequence of the human genome. Science.

[12] Liu S., da Cunha A.P., Rezende R.M., Cialic R., Wei Z., Bry L., Comstock L.E., Gandhi R., Weiner H.L. (2016). The host shapes the gut microbiota via fecal microRNA. Cell Host Microb..

[13] Arumugam M., Raes J., Pelletier E., Le Paslier D., Yamada T., Mende D.R., Fernandes G.R., Tap J., Bruls T., Batto J.-M. (2011). Enterotypes of the human gut microbiome. Nature.

[14] Wu G.D., Chen J., Hoffmann C., Bittinger K., Chen Y.Y., Keilbaugh S.A., Bewtra M., Knights D., Walters W.A., Knight R. (2011). Linking long-term dietary patterns with gut microbial enterotypes. Science.

[15] Ma H., Zhang B., Hu Y., Wang J., Liu J., Qin R., Lv S., Wang S. (2019). Correlation analysis of intestinal redox state with the gut microbiota reveals the positive intervention of tea polyphenols on hyperlipidemia in high fat diet fed mice. J. Agric. Food Chem..

[16] Kong C., Gao R., Yan X., Huang L., Qin H. (2019). Probiotics improve gut microbiota dysbiosis in obese mice fed a high-fat or high-sucrose diet. Nutrition.

[17] An Y., Li Y., Wang X., Chen Z., Xu H., Wu L. (2018). Cordycepin reduces weight through regulating gut microbiota in high-fat diet-induced obese rats. Lipids Health Dis..

[18] Chen L., Zhang L., Wang W., Qiu W., Liu L., Ning A., Cao J., Huang M., Zhong M. (2020). Polysaccharides isolated from *Cordyceps sinensis* contribute to the progression of NASH by modifying the gut microbiota in mice fed a high-fat diet. PLoS ONE.

[19] Chen S., Wang J., Fang Q., Dong N., Nie S. (2019). Polysaccharide from natural *Cordyceps sinensis* ameliorated intestinal injury and enhanced antioxidant activity in immunosuppressed mice. Food Hydrocoll..

[20] Wang J., Nie S., Cui S.W., Wang Z., Phillips A.O., Phillips G.O., Li Y., Xie M. (2017). Structural characterization and immunostimulatory activity of a glucan from natural *Cordyceps sinensis*. Food Hydrocoll..

[21] Wang J., Nie S., Chen S., Phillips A.O., Phillips G.O., Li Y., Xie M., Cui S.W. (2018). Structural characterization of an α-1,6-linked galactomannan from natural Cordyceps sinensis. Food Hydrocoll..

[22] Kong X.R., Zhu Z.Y., Zhang X.J., Zhu Y.M. (2020). Effects of *Cordyceps* polysaccharides on pasting properties and in vitro starch digestibility of wheat starch. Food Hydrocoll..

[23] Chang C.Y., Lue M.Y., Pan T.M. (2005). Determination of adenosine, cordycepin and ergosterol contents in cultivated *Antrodia camphorate* by HPLC methods. J. Food Drug Anal..

[24] Urtasun R., Diaz-Gomez J., Arana M., Pajares M.J., Oneca M., Torre P., Jimenez M., Munilla G., Varajas M., Encio I. (2020). A combination of apple vinegar drink with *Bacillus coagulans* ameliorates high fat diet-induced body weight gain, insulin resistance and hepatic steatosis. Nutrients.

[25] Liu P., Xie J., Liu J., Ouyang J. (2019). A novel thermostable β-galactosidase from *Bacillus coagulans* with excellent hydrolysis ability for lactose in whey. J. Dairy Sci..

[26] Jia J., Zhang X., Hu Y.S., Wu Y., Wang Q.Z., Li N.N., Guo Q.C., Dong X.C. (2009). Evaluation of in vivo antioxidant activities of *Ganoderma lucidum* polysaccharides in STZ-diabetic rats. Food Chem..

[27] Dall’Agnol R., Von Poser G.L. (2000). The use of complex polysaccharides in the management of metabolic diseases: The case of *Solanum lycocarpum* fruits. J. Ethnopharmacol..

[28] Lee B.H., Hsu W.H., Pan T.M. (2011). Inhibitory effects of dioscorea polysaccharide on TNF-α-induced insulin resistance in mouse FL83B cells. J. Agric. Food Chem..

[29] Li M.M. (2007). Protective effect of *Lycium barbarum* polysaccharides on streptozotocin-induced oxidative stress in rats. Int. J. Biol. Macromol..

[30] Nava G.M., Friedrichsen H.J., Stappenbeck T.S. (2011). Spatial organization of intestinal microbiota in the mouse ascending colon. ISME J..

[31] Skoog E.C., Lindberg M., Linden S.K. (2011). Strain-dependent proliferation in response to human gastric mucin and adhesion properties of *Helicobacter pylori* are not affected by co-isolated *Lactobacillus* sp.. Helicobacter.

[32] Bai Z., Zhang Z., Ye Y., Wang S. (2010). Sodium butyrate induces differentiation of gastric cancer cells to intestinal cells via the PTEN/phosphoinositide 3-kinase pathway. Cell Biol. Int..

[33] Crost E.H., Tailford L.E., Monestier M., Swarbreck D., Henrissat B., Crossman L.C., Juge N. (2016). The mucin-degradation strategy of *Ruminococcus gnavus*: The importance of intramolecular trans-sialidases. Gut Microbes.

[34] Derrien M., Vaughan E.E., Plugge C.M., de Vos W.M. (2004). *Akkermansia muciniphila* gen. nov., sp. nov., a human intestinal mucin-degrading bacterium. Int. J. Syst. Evolut. Microbiol..

[35] Xu J., Bjursell M.K., Himrod J., Deng S., Carmichael L.K., Chiang H.C., Hooper L.V., Gordon J.I. (2003). A genomic view of the human-*Bacteroides thetaiotaomicron* symbiosis. Science.

[36] He F., Ouwehan A.C., Hashimoto H., Isolauri E., Benno Y., Salminen S. (2001). Adhesion of *Bifidobacterium* spp. to human intestinal mucus. Microbiol. Immunol..

[37] Macfarlane G.T., Gibson G.R. (1991). Formation of glycoprotein degrading enzymes by *Bacteroides fragilis*. FEMS Microbiol. Lett..

[38] Png C.W., Linden S.K., Gilshenan K.S., Zoetendal E.G., McSweeney C.S., Sly L.I. (2010). Mucolytic bacteria with increased prevalence in IBD mucosa augment in vitro utilization of mucin by other bacteria. Am. J. Gastroenterol..

[39] Berry D., Stecher B., Schintlmeister A., Reichert J., Brugiroux S., Wild B. (2013). Host-compound foraging by intestinal microbiota revealed by single-cell stable isotope probing. Proc. Natl. Acad. Sci. USA.

[40] Van den Abbeele P., Belzer C., Goossens M., Kleerebezem M., De Vos W.M., Thas O. (2013). Butyrate-producing *Clostridium* cluster XIVa species specifically colonize mucins in an in vitro gut model. ISME J..

[41] Ashida H., Maki R., Ozawa H., Tani Y., Kiyohara M., Fujita M. (2008). Characterization of two different endo-alpha-N-acetylgalactosaminidases from probiotic and pathogenic enterobacteria, *Bifidobacterium longum* and *Clostridium perfringens*. Glycobiology.

[42] Ng K.M., Ferreyra J.A., Higginbottom S.K., Lynch J.B., Kashyap P.C., Gopinath S. (2013). Microbiota-liberated host sugars facilitate post-antibiotic expansion of enteric pathogens. Nature.

[43] Etzold S., Kober O.I., MacKenzie D.A., Tailford L.E., Gunning A.P., Walshaw J. (2014). Structural basis for adaptation of lactobacilli to gastrointestinal mucus. Environ. Microbiol..

[44] Sicard J.F., Le Bihan G., Vogeleer P., Jacques M., Harel J. (2017). Interactions of intestinal bacteria with components of the intestinal mucus. Front. Cell. Infect. Microbiol..

[45] Clarke K.R. (1993). Non-parametric multivariate analysis of changes in community structure. Aust. J. Ecol..

[46] Segata N., Izard J., Waldron L., Gevers D., Miropolsky L., Garrett W.S., Huttenhower C. (2011). Metagenomic biomarker discovery and explanation. Genome Biol..

[47] Cani P.D., de Vos W.M. (2017). Next-generation beneficial microbes: The case of *Akkermansia muciniphila*. Front. Microbiol..

[48] Thomas L.V., Suzuki K., Zhao J. (2015). Probiotics: A proactive approach to health. A symposium report. Br. J. Nutr..

[49] Shih C.T., Yeh Y.T., Lin C.C., Yang L.Y., Chiang C.P. (2020). *Akkermansia muciniphila* is negatively correlated with hemoglobin A1c in refractory diabetes. Microorganisms.

[50] Dao M.C., Everard A., Aron-Wisnewsky J., Sokolovska N., Prifti E., Verger E.O., Kayser B.D., Levenez F., Chilloux J., Hoyles L. (2016). *Akkermansia muciniphila* and improved metabolic health during a dietary intervention in obesity: Relationship with gut microbiome richness and ecology. Gut.

[51] Lee B.H., Lo Y.H., Pan T.M. (2013). Anti-obesity activity of *Lactobacillus* fermented soy milk products. J. Funct. Foods.

[52] Lippert K., Kedenko L., Antonielli L., Kedenko I., Gemeier C., Leitner M., Kautzky-Willer A., Paulweber B., Hacki E. (2017). Gut microbiota dysbiosis associated with glucose metabolism disorders and the metabolic syndrome in older adults. Benef. Microbes.

[53] Kasahara K., Krautkramer K.A., Org E., Romano K.A., Kerby R.L., Vivas E.I., Mehrabian M., Denu J.M., Backhed F., Lusis A. (2018). Interactions between *Roseburia* intestinalis and diet modulate atherogenesis in a murine model. Nat. Microbiol..

[54] Chen W., Liu F., Ling Z., Tong X., Xiang C. (2012). Human intestinal lumen and mucosa-associated microbiota in patients with colorectal cancer. PLoS ONE.

[55] Bajaj J.S., Hylemon P.B., Ridlon J.M., Heuman D.M., Daita K., White M.B., Monteith P., Noble N.A., Sikaroodi M.S., Gillevet P.M. (2012). Colonic mucosal microbiome differs from stool microbiome in cirrhosis and hepatic encephalopathy and is linked to cognition and inflammation. Am. J. Physiol. Gastrointest. Liver Physiol..

[56] Schirmer M., Smeekens S.P., Vlamakis H., Jaeger M., Oosting M., Franzosa E.A., Horst R.T., Jansen T., Jacobs L., Bonder M.J. (2016). Linking the human gut microbiome to inflammatory cytokine production capacity. Cell.

[57] Shen F., Zheng R.D., Sun X.Q., Ding W.J., Wang X.Y., Fan J.G. (2017). Gut microbiota dysbiosis in patients with non-alcoholic fatty liver disease. Hepat. Pancreat. Dis. Int..

